# Identifying 1q amplification and PHF19 expressing high-risk cells associated with relapsed/refractory multiple myeloma

**DOI:** 10.21203/rs.3.rs-3221549/v1

**Published:** 2023-08-16

**Authors:** Travis S. Johnson, Parvathi Sudha, Enze Liu, Patrick Blaney, Gareth Morgan, Vivek S. Chopra, Cedric Dos Santos, Michael Nixon, Kun Huang, Attaya Suvannasankha, Mohammad Abu Zaid, Rafat Abonour, Brian A. Walker

**Affiliations:** 1 Department of Biostatistics and Health Data Science, School of Medicine, Indiana University, Indianapolis, IN, USA; 2 Indiana Biosciences Research Institute, Indianapolis, IN, USA; 3 Melvin and Bren Simon Comprehensive Cancer Center, Experimental and Developmental Therapeutics, School of Medicine, Indiana University, Indianapolis, IN, USA; 4 Center for Computational Biology and Bioinformatics, School of Medicine, Indiana University, Indianapolis, IN, USA; 5 Melvin and Bren Simon Comprehensive Cancer Center, Division of Hematology and Oncology, School of Medicine, Indiana University, Indianapolis, IN, USA; 6 Perlmutter Cancer Center, Langone Health, New York University, New York, NY, USA; 7 Genentech Inc., South San Francisco, CA, USA; 8 Roche Inc., Indianapolis, IN, USA; 9 Roudebush VAMC, Indianapolis, IN, USA

## Abstract

Multiple Myeloma is an incurable plasma cell malignancy with a poor survival rate that is usually treated with immunomodulatory drugs (iMiDs) and proteosome inhibitors (PIs). The malignant plasma cells quickly become resistant to these agents causing relapse and uncontrolled growth of resistant clones. From whole genome sequencing (WGS) and RNA sequencing (RNA-seq) studies, different high-risk translocation, copy number, mutational, and transcriptional markers have been identified. One of these markers, *PHF19*, epigenetically regulates cell cycle and other processes and has already been studied using RNA-seq. In this study a massive (325,025 cells and 49 patients) single cell multiomic dataset was generated with jointly quantified ATAC- and RNA-seq for each cell and matched genomic profiles for each patient. We identified an association between one plasma cell subtype with myeloma progression that we have called relapsed/refractory plasma cells (RRPCs). These cells are associated with 1q alterations, TP53 mutations, and higher expression of *PHF19*. We also identified downstream regulation of cell cycle inhibitors in these cells, possible regulation of the transcription factor (TF) PBX1 on 1q, and determined that *PHF19* may be acting primarily through this subset of cells.

## INTRODUCTION

Myeloma is a plasma cell malignancy with a poor survival rate that in current years has seen improved prognosis due to advances in treatment.^1^ Disease progression and more generally disease risk, are defined by molecular subtypes of myeloma.^2^ Currently there are multiple genomic markers that impact patient survival and likelihood to relapse^3,4^ such as t(4;14)^5^, Del17p^3,6^, or Gain/Amp1q^7,8^ which have been long associated with worse prognosis. Additional copies of chromosome 1 (Gain/Amp1q) are the most common chromosomal abnormalities in MM. Recently, Gain/Amp1q and especially Amp1q have emerged as a poor risk factor including risk of relapse with the current frontline therapies for myeloma.^9^ Despite this attention, the mechanism through which Gain/Amp1q affects prognosis and response to therapy is still not known. Some hypotheses for the Gain/Amp1q mechanism include increased expression of *CKS1B* affecting *SKP2* and *KIP27*^10^ or more recently the transcription factor (TF), *PBX1*, affecting *FOXM1*^11^. For these reasons there is still a need to understand the mechanisms through which Gain/Amp1q leads to poor prognosis.

Besides translocations and copy number variations that affect myeloma disease progression, there are many epigenetic regulators that affect chromatin accessibility and result in altered protein expression and eventually progression.^12^ PHD finger protein 19 (PHF19) is an epigenetic regulator that has recently seen much interest in the myeloma field due to its relationship with poor patient outcomes.^13^ It has been shown that PHF19 can affect cell cycle by interacting with the polycomb repressive complex 2 (PRC2) to induce trimethylation of histone H3 lysine 27 (H3K27me3).^14^ Over-expression of *PHF19* has been shown to increase H3K27me3 at cell cycle inhibitor and JAK-STAT loci in the genome.^14^ Though these regulatory relationships have been evaluated using cell line experiments and using patient-level omics datasets there has yet to be an evaluation of these mechanisms at the single-cell level.

Recently, single cell RNA sequencing datasets have been generated from cohorts of myeloma patients. Initially, these datasets showed that there were key differences between asymptomatic multiple myeloma patients and symptomatic multiple myeloma patients.^15^ Both in patients with asymptomatic multiple myeloma and patients post treatment with minimum residual disease there were detectable levels of rare tumor cells with characteristics of active myeloma.^15^ Subsequently, relapsed and refractory patients after bortezomib treatment acquired new resistance mechanisms including hypoxia tolerance, protein folding, and mitochondria respiration.^16^ Aside from noticeable changes in the tumor cell fraction that improve resistance to treatment and more aggressive proliferation, the immune microenvironment is compromised in earlier precursors to multiple myeloma.^17^ All of these studies show that the complex interplay between the tumor and immune microenvironments are largely dysregulated even in early stages of disease. These changes can sometimes be attributed to epigenetic regulatory mechanisms.^12^

In this study, we use a large single-cell multiomic dataset to show how cytogenetic changes affect the progression of myeloma. Using chromatin accessibility paired with gene expression we were able to recapitulate epigenetic regulation of the cell cycle in human samples at the single-cell level. We were able to link these epigenetic relationships back to cytogenetic events uncovering new mechanisms through which genomic events lead to increased cell proliferation.

## METHODS

### Sample collection and sequencing

Bone marrow aspirates from 10 smoldering multiple myeloma (SMM), 22 newly diagnosed multiple myeloma (NDMM), and 17 relapsed or refractory multiple myeloma (RRMM) patients were collected through the Indiana Myeloma Registry (Table 1). These samples underwent CD138+ magnetic-activated cell sorting resulting in a CD138+ fraction with average purity 84.0 ± 9.6% and were viably frozen.

### Single-cell multiomic sequencing

Single-cell multiome analysis was conducted using a 10X Chromium system (10X Genomics, Inc). Cryopreserved cells were thawed and washed as previously described.^18^ The cell suspensions were processed into RNA and ATAC libraries using the manufacturers standard protocol (**Supplementary Materials**). The resulting ATAC and cDNA libraries were sequenced separately, cDNA library for 28 bp and 91 bp paired-end and ATAC library for 50 bp paired-end sequencing on a NovaSeq 6000 (Illumina).

### Bulk DNA sequencing

CD138+ cells for 44 samples also underwent bulk whole genome sequencing (WGS) and targeted panel sequencing^19^ to identify copy number alterations, translocations, and single nucleotide variations. For WGS, genomic DNA from tumor and non-tumor (saliva or peripheral blood sample from the same patient) samples were prepared using the DNA PCR-free Library Prep Tagmentation Kit (Illumina). Libraries were pooled and sequenced in 150 bp paired-end read format on a NovaSeq 6000 (Illumina) to a mean depth of 73x for tumor samples and 27x for matched control samples.

WGS samples were pre-processed using the Myeloma Genome Project 1000 (MGP1000) pipeline (https://github.com/pblaney/mgp1000). Coverage metrics and GC bias metrics were calculated for each BAM file. For each matched normal/tumor pair, genetic concordance and contaminations were estimated using Conpair (v0.2)^20^ prior to variant analysis. Strelka (v.2.9.2)^21^ was used for variant calling and single nucleotide variants (SNVs) were filtered using fpfilter (https://github.com/ckandoth/variant-filter) to a 5% VAF cut-off.^22^ Indels were filtered using a 10% VAF cut-off. Variants were annotated using Variant Effect Predictor (v.101).^23^ Structural variants were calculated using Manta (v1.6.0).^24^ Copy number variations were analyzed using ASCAT-NGS.^25^

### Cleaning and Preprocessing of CD138+ scRNA-seq

**Fig. 1:**
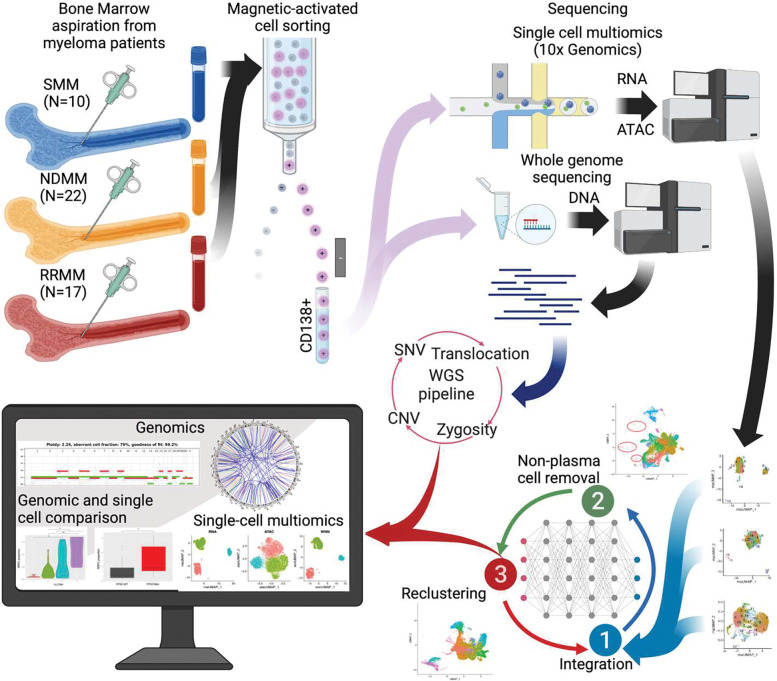
Overview of the samples collection, processing, and our analysis pipelines for WGS and single cell multiomics.

The single-cell multiomics reads for the CD138+ fraction were aligned and quantified using the cellranger-arc software (10X Genomics). These aligned and quantified reads were used as input to a custom-built Seurat pipeline for single-cell multiomics that performed quality control (QC), multi-dataset integration, and non-plasma cell removal (Fig. 1). For each dataset, QC was performed including removal of: low expression cells, cells with low number of features, cells with high number of features, and cells with high percentage mitochondrial RNA as outlined in Seurat documentation and online resources^26,27^ (**Supplementary Materials**).

### Integration of scRNA-seq across samples and non-plasma cell removal

An iterative process was used to remove non-plasma cells from the dataset (Fig. 1). In the first iteration, all 49 samples were integrated together based on their RNA profiles using Seurat v4. The expression of *SDC1* (CD138) was summarized for each cluster using percentiles such that the 10^th^, 25^th^, 50^th^, 75^th^, and 90^th^ percentile cells were used to summarize the expression in a given cluster. Clusters with *SDC1* expression with 0 counts in the 90^th^ percentile cell were removed as contaminant cells. After contaminant cell removal, a second iteration of integration was performed on the remaining cells. In this iteration, clusters were removed if *SDC1* expression was 0 counts in the 75^th^ percentile cell of that cluster. The remaining clusters were used for the remainder of the analysis to study changes in myeloma cells across stages of progression. Samples were annotated based on the WGS data for common CNVs and translocations, which were confirmed by expression of translocation markers (*NSD2, FGFR3, CCND1, CCND3, MAF, MAFB, ITGB7, CCND2, MYC*) in the single-cell data.

### Identification of high-risk myeloma cells from scRNA-seq

For each of the identified plasma cell clusters derived from the CD138+ samples, the proportion of that cluster was calculated for each patient such that each patient had a total proportion of 1.0 split between each individual cluster. The cluster proportions across all of the patients were compared to high-risk copy number alterations, disease status, and mutation events to identify clusters associated with these variables. For high-risk clusters, differentially expressed gene (DEG) analysis was conducted on the RNA-seq and differentially accessible chromatin (DAC) analysis on the ATAC-seq. Firstly, the ATAC-seq data was aggregated to the gene level using the gene activity estimates provided by the Seurat package. The DEG and DAC analyses were conducted by calculating the log_2_FC and Wilcoxon test p-value for each gene. The Benjamini-Hochberg correction was used for multiple testing correction of the p-values to false discover rate (BH-FDR). For the DEG analysis, an absolute log_2_FC value of >1.5 and BH-FDR of <0.05 were used as cutoffs. For the DAC analysis, an absolute log_2_FC value of >1.25 and BH-FDR of <0.05 were used as cutoffs.

Besides the DEGs and DACs identified from our own data, DEGs were also used from previous *PHF19* knock down (KD) experiments^14^ from the differential expression table for the *PHF19* KD and *PHF19* rescue experiments including the log_2_FC, p-values, and adjusted p-values with a fold change cutoff (FC=2) and a p-value cutoff of 0.05. Besides the *PHF19* KD signature, previously published proliferation signatures^28,2^ were also included for comparison purposes. The intersections of DEGs, DACs, *PHF19* KO DEGs, and other known myeloma signatures were used to filter the genes into high-risk subsets for further study.

### Individual multiomic integration and inference of CNVs at the single-cell level

Aside from the genomic alterations that were measured from WGS, it was also important to evaluate the subclonal structure within each sample and to impute samples that were missing WGS information (**Supplementary Materials**). The subclonal structure may be reflected in both the RNA-seq, ATAC-seq, and integrated clusters. For these reasons multiomic integration was performed for each sample using Seurat v4. Specifically, for each sample, the ATAC-seq count matrices and RNA-seq count matrices were loaded into Seurat and converted into a Seurat object where the same non-plasma cell remove process was performed as in the original 49-sample integration for each sample individually. The ATAC-seq and RNA-seq data for these plasma cell clusters were integrated using the weighted nearest neighbor (WNN) algorithm from the Seurat package. These WNN-clusters were used as our clustering variable for single cell CNV inference. For this analysis, the package inferCNV^29^ was used to identify CNVs at the single cell level. Based on the previous analysis of plasma cell clusters, the normal plasma cell cluster that was identified was used as the reference for inferCNV. Single cell CNVs were also calculated using a reference free software package, CopyKAT^30^, to get location specific CNVs instead of gene specific. From these analyses the CNVs that correspond to clustering in the RNA-seq and ATAC-seq data were evaluated.

### Identification of *PHF19* promoter binding transcription factors (TF) and TF ChIP-seq peaks

Based on the preliminary analyses, the relationship between *PHF19* and high-risk copy number alterations that may contain TFs were evaluated. The TF databases, hTFtarget^31^ and TF2DNA^32^ were used to identify TFs that regulate *PHF19*. The identified *PHF19*-regulating TFs were cross-referenced against high-risk CNVs in myeloma. Paired ChIP-seq and RNA-seq in myeloma^11^ were used to evaluate whether there were ChIP peaks upstream of *PHF19*. Both the existence of peaks within 5000 bp of the 5’ UTR as well as their relative strength in comparison to the other peaks across the genome were used to evaluate whether they likely indicate TF regulation of *PHF19*. Specifically, all of the identified peaks in the ChIP-seq experiments were ranked and the percentile of the peaks upstream of *PHF19* was used to discern how likely the TF was regulating *PHF19* opposed to the other peaks.

## RESULTS

### Integrated clustering identifies cells associated with relapse and high-risk markers

**Fig. 2:**
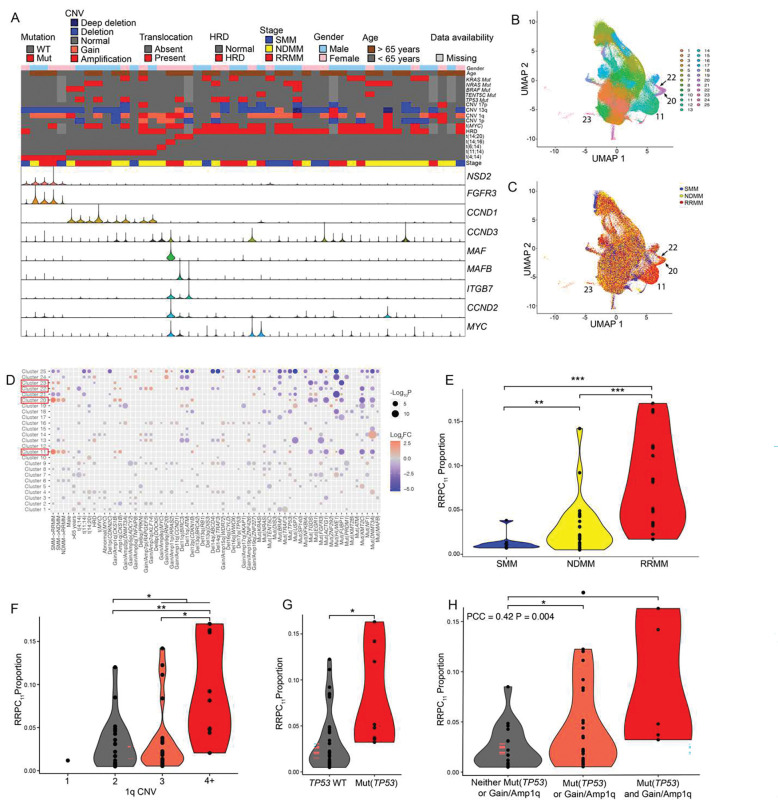
Characterization and integration of plasma cell clusters. **A)** Canonical translocations, mutations, and copy number alteration markers in each patient **B)** Final clusters determined from the dataset integration of 49 samples. **C)** Diagnosis of the patient from which each cell was derived overlaid on to the integrated clusters. **D)** Significant associations between clusters and covariates (clinical and genomic) **E)** Proportion of RRPCs in patients stratified by diagnosis. **F)** Proportion of RRPC_11_ in patients stratified by 1q copy number. **G)** Proportion of RRPC_11_ in patients stratified by *TP53* mutation status. **H)** Proportion of RRPC_11_ in patients with gain/Amp1q, *TP53* mutations, or both. Significance levels are: P<0.1: (·), P<0.05: *, P<0.01 (**), P<0.001 (***).

CD138+ single cells were assayed by multiome sequencing (10X genomics) from 49 patients with SMM (n=10), NDMM (n=22), and RRMM (n=17). Samples initially had an average of 9484 cells. Removal of low-quality cells and non-plasma cells resulted in 6819 cells per sample for downstream analyses (**Fig. S1**). Based on our analysis pipeline to integrate all patient samples, 325,025 high quality myeloma cells were retained for further study. These cells were used to impute translocations: t(4;14), t(6;14), t(11;14), t(14;16), and t(14;20) (**Fig. S2**), CNVs: 1p, 1q, 13q, and 17p (**Fig. S3**), and HRD (**Fig. S4**) in the five patient samples missing WGS (**Table S1**). Of the patients, 19 had a translocation that could be stratified into the primary translocation groups based on marker gene expression (Fig. 2A).

Cells were integrated and formed 25 distinct clusters (Fig. 2B) of myeloma cells with unique transcriptome profiles that frequently correlated with stage of progression (Fig. 2C) and genomic events (Fig. 2D). Using the defined clusters, we determined if any were associated with disease stage, or cytogenetic subgroup (Fig. 2D). We determined that clusters 11, 20, 22, and 23 were associated with later stages of myeloma progression (Fig. 2B–D) such that all of these clusters had significantly greater proportions in RRMM compared to SMM (P<0.001, P<0.001, P=0.031, and P=0.032, respectively). The largest of these clusters, cluster 11, increased in proportion from SMM to NDMM (1% to 3%, P=0.008, Fig. 2E), and NDMM to RRMM (3% to 8%, P<0.001, Fig. 2E). Due to their greater enrichment in RRMM patients in comparison to earlier stages of myeloma we denote clusters 11, 20, 22, and 23 as relapse/refractory plasma cells (RRPC) or RRPC_11_, RRPC_20_, RRPC_22_, and RRPC_23_ for individual RRPC clusters (Table 3).

Aside from a clear association of RRPCs with myeloma progression, there were also associations between the proportions of these cells and high-risk genomic events (**Table S2**, Fig. 2D). The proportion of all RRPCs was associated with 1q copy number such that samples with a normal copy number of 1q had a significantly lower proportion of RRPCs compared to Gain/Amp1q samples (4% vs. 8%, P=0.019; Fig. 2D). Patients with Gain/Amp1q (defined by WGS) had significantly more cells in cluster RRPC_11_ than those with a normal copy number of 1q (0.05 vs. 0.03, P = 0.030; Fig. 2F) and those with Amp1q had significantly more RRPC_11_ than those with Gain1q (4% vs. 9%, P=0.014; Fig. 2F). In addition, samples with a *TP53* mutation had a significantly greater proportion of RRPC_11_ than samples without a *TP53* mutation (0.08 vs. 0.03, P = 0.025; Fig. 2G). There was also a synergistic effect between *TP53* mutation status and Gain/Amp1q such that the number of these alterations was correlated with RRPC_11_ proportion (PCC=0.42, P=0.004, Fig. 2H). Additionally, Del12p(*CDKN1B*), *NRAS* mutations, and HUWE1 mutations were also associated with RRPC_11_. Although clusters RRPC_11_, RRPC_20_, RRPC_22_, and RRPC_23_ were all significantly associated with Gain/Amp1q (Fig. 2D, Table 3), only RRPC_11_, RRPC_20_, and RRPC_22_ were associated with Amp1q (Fig. 2D) and adjacently clustered to each other (Fig. 2B+C). RRPC clusters were not enriched for other high-risk markers such as Del(*CDKN2C*), t(4;14), or t(14;16). RRPC_11_ was also the most prevalent of the RRPC clusters. All of these factors make RRPCs and more specifically RRPC_11_ an important group of cells for further study.

### RRPCs have increased expression of the epigenetic modifier *PHF19* and proliferative genes

**Fig. 3:**
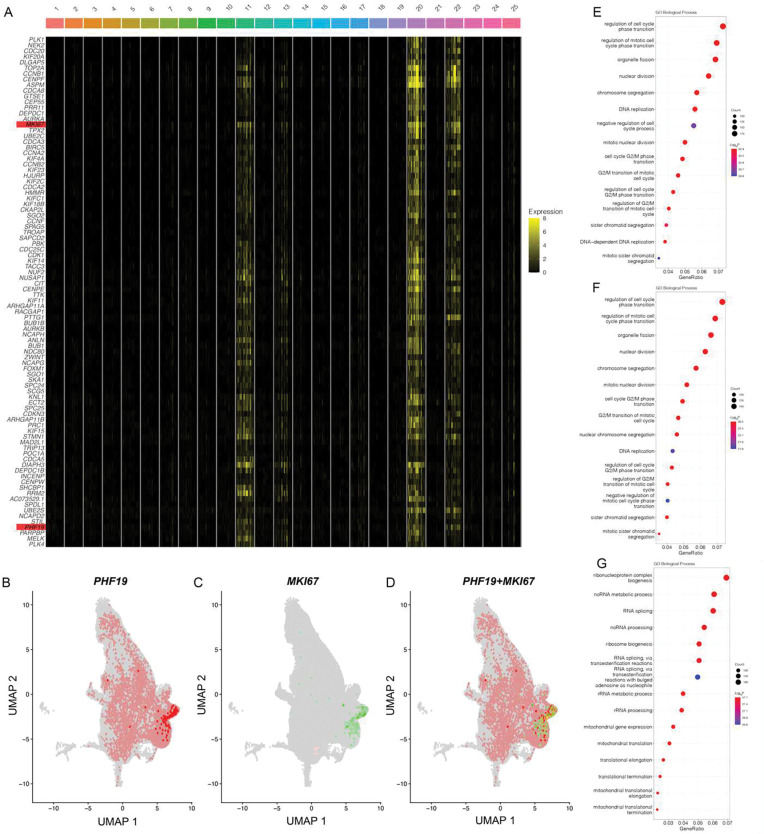
Differential expression for the top intersecting RRPC_11_, RRPC_20_, and RRPC_22_ markers. **A)** Heatmap of the top 90 up-regulated DEGs and top 90 downregulated DEGs common between RRPC_11_, RRPC_20_, and RRPC_22_. **B-D)** RRPC clusters coexpress *PHF19* and *MKI67*. **B)** *PHF19* expression in integrated dataset. **C)** *MKI67* expression in integrated dataset. **D)** Co-expression of *PHF19* and *MKI67* in integrated dataset. **E-G)** Top 15 significant GO Biological Process for RRPC_11_ DEGs **(E)**, RRPC_20_ DEGs **(F)**, and RRPC_22_ DEGs **(G)**.

To better understand the function of RRPCs, marker genes were identified using differential gene expression analysis comparing each RRPC cluster to all other clusters. For RRPC_11_, this identified 8232 up-regulated and 1590 down-regulated DEGs (**Table S3**) and many were also differentially expressed in both RRPC_20_ and RRPC_22_ (1247 up-regulated and 191 down-regulated common genes, **Table S4, S5**). RRPC_11,20,22_ all had similar expression profiles (Fig. 3A). Notably, RRPC_11,20,22_ had greater expression of *PHF19* than other clusters (RRPC_11_: log_2_FC=2.43, P<0.001; RRPC_20_: log_2_FC =3.08, P<0.001; RRPC_22_: log_2_FC=2.77, P<0.001, Fig. 3A+B), an epigenetic modifier whose expression is highly predictive of poor prognosis in myeloma patients.^13^ One of the main markers for proliferation, *MKI67* is also highly expressed in RRPC_11,20,22_ (RRPC_11_: log_2_FC=3.78, P<0.001; RRPC_20_: log_2_FC=4.37, P<0.001; RRPC_22_: log_2_FC=3.80, P<0.001, Fig. 3A,C) and co-expressed with *PHF19* (Fig. 3D). Furthermore, we see enrichment for cell-cycle related processes in RRPC_11_ (Fig. 3E), RRPC_20_ (Fig. 3F), and RRPC_22_ (Fig. 3G). *PHF19* has been shown to negatively affect the expression of cell cycle inhibitors, therefore promoting proliferation.^14^ Taking into consideration the association of RRPC clusters with gain/amp1q, *TP53* mutations, and increased expression of *PHF19*, RRPC clusters and especially RRPC_11,20,22_ should be considered a high-risk subset of cells.

### RRPCs have reduced expression of cell-cycle inhibitors associated with loss of chromatin accessibility.

**Fig. 4:**
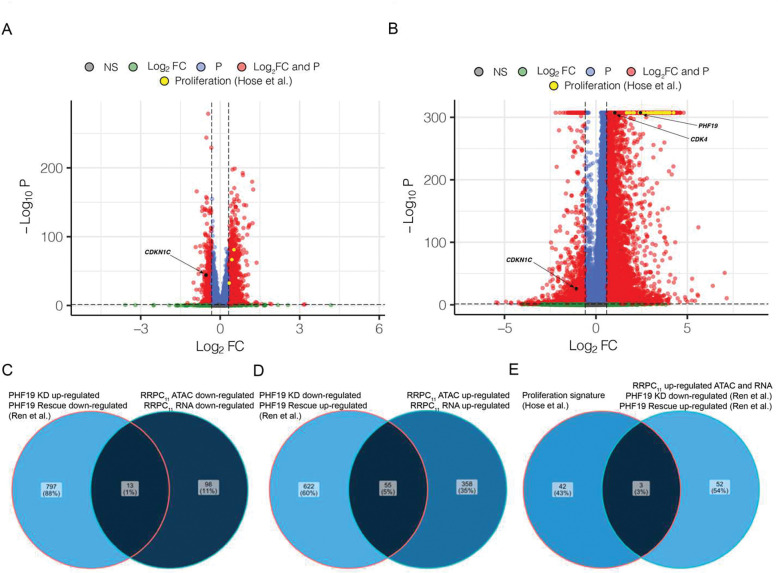
Differential gene expression and chromatin accessibility in RRPC_11_. **A)** Gene activity differential chromatin accessibility for RRPC_11_ with proliferation genes annotated. **B)** Differentially expressed genes for RRPC_11_ with proliferation genes annotated. **C)** Intersection of PHF19 repressed genes from Ren et al. versus RRPC_11_ ATAC and RNA downregulated genes. **D)** Intersection of PHF19 up-regulated genes from Ren et al. versus RRPC_11_ ATAC and RNA up-regulated genes. **E)** Intersection of **(D)** with proliferation signature genes.

Given that PHF19 is known to regulate negative regulators of cell cycle^14^ we analyzed both the scRNA-seq and scATAC-seq data for changes in cell cycle and proliferation genes.^28^ Compared to other clusters, proliferative index genes had increased chromatin accessibility (Fig. 4A) and increased expression (Fig. 4B) in the RRPC_11_ cluster. In RRPC_11_, *PHF19* was significantly up-regulated (log_2_FC=2.43, P<0.001, Fig. 4B), *CDKN1C* had reduced chromatin accessibility (log_2_FC=−0.54, P<0.001, Fig. 4A) and reduced expression (log_2_FC=−1.08, P<0.001, Fig. 4B), and *CDK4* had increased expression (log_2_FC=1.02, P<0.0001, Fig. 4B) compared to all other clusters. Since, previous studies have already established epigenetic regulatory mechanisms of *CDKN1C* by PHF19,^14^ these results demonstrate that a subset of proliferative cells with high *PHF19* expression, found primarily in relapsed or refractory patients, also likely epigenetically downregulate *CDKN1C* (Fig. 4A+B).

To further verify these patterns, data from cell lines where *PHF19* was knocked-down and subsequently rescued was analyzed.^14^ A significant overlap between genes that were up-regulated from the *PHF19* knockdown experiments and genes downregulated in both the ATAC-seq and RNA-seq from RRPC_11_ was found (OR=2.24, P=0.010, Fig. 4C). Similarly, the genes that were down-regulated by *PHF19* knockdown had significant overlap with genes found to be upregulated in both ATAC-seq and RNA-seq from RRPC_11_ (OR=3.30, P<0.001, Fig. 4D). Upon further examination of the genes which were up-regulated in both ATAC-seq and RNA-seq, three were also in a known myeloma proliferation signature,^28^ namely: *NEK2*, *AURKB*, and *CCNB2* (Fig. 4E). These genes also represent potential therapeutic targets for relapsed/refractory patients who have had multiple failed treatments that would target the high risk RRPC_11_ cluster.

### PBX1 on 1q regulates expression of *PHF19* in RRPCs

**Fig. 5:**
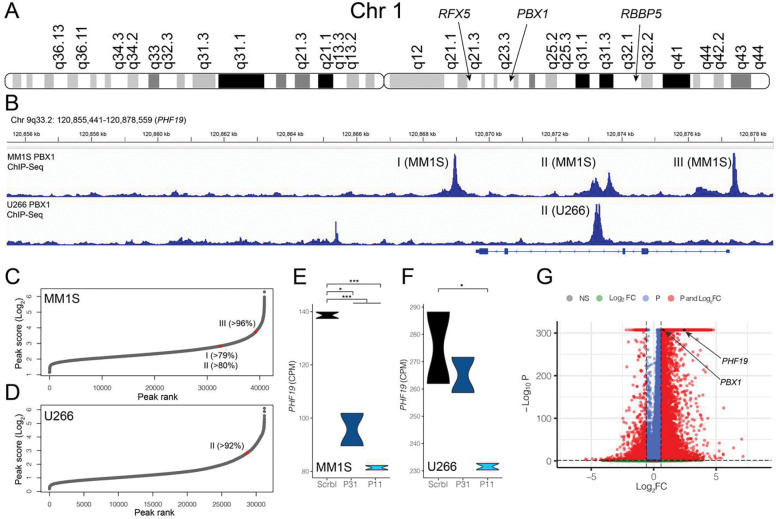
Association of the chromosome 1q TFs with *PHF19* expression. **A)** Location of TFs on chromosome 1q. **B)** PBX1 ChIP-seq peaks upstream of *PHF19* on chromosome 9. **C-D)** PBX1 ChIP-seq peak scores in MM1S **(C)** and U266 **(D)** myeloma cell lines with the percentile of each ChiP-seq peak marked. **E)** Differences in *PHF19* expression in MM1S cells treated with PBX1 shRNAs (P11 and P31). **F)** Differences in *PHF19* expression in U266 cells treated with PBX1 shRNAs (P11 and P31). **G)** Differential expression of *PBX1* and *PHF19* in RRPCs. Significance levels are: P<0.1: (·), P<0.05: *, P<0.01 (**), P<0.001 (***).

To further evaluate the connections between copy number variations and regulation of *PHF19* expression, transcription factors (TFs) were identified that were located on regions of the genome that were amplified (> 3 copies) in patients with higher proportions of RRPCs. The only significant gain or amplification affecting all RRPC cluster proportions was Gain/Amp1q (Fig. 2D, Table 3). The TF2DNA^32^ and hTFtarget^31^ databases contain pairs of TFs and their potential targets. In total, 35 TFs were identified from TF2DNA and 57 TFs were identified from hTFtarget that target *PHF19*. Of these TFs, seven (*ATF3*, *KDM5B*, *PBX1*, *RBBP5*, *RFX5*, *USF1*, *ZNF648*) were located on 1q, of which *PBX1* (log_2_FC=0.78, P <0.001), *RFX5* (log_2_FC=0.81, P<0.001), and *RBBP5* (log_2_FC=0.93, P<0.001) were significantly up-regulated in RRPC_11_ compared to all other clusters (Fig. 5A). PBX1 ChIP-seq and RNA-seq data was available^11^ and was used to investigate PBX1 binding in myeloma cell lines. From the myeloma cell lines MM1S and U266, both of which have Gain/Amp1q, ChIP-seq peaks were identified in the promoter of *PHF19* that were in the top 4% of MM1S ChIP-seq peaks and the top 8% of U266 ChIP-seq peaks (Fig. 5B–D). From RNA-seq data of MM1S (Fig. 5E) and U266 (Fig. 5F), silencing *PBX1* via shRNA significantly decreased *PHF19* expression. Notably, both *PHF19* (log_2_FC=2.43 P<0.0001) and *PBX1* (log_2_FC=0.78, P <0.0001) are upregulated in RRPC_11_ (Fig. 5G). *PHF19* expression is detectable in 47% of RRPC_11_ cells compared to only 9% of cells in other clusters and *PBX1* expression is detectable in 22% of RRPC_11_ compared to only 10% of cells in other clusters, further strengthening their biological link.

### Analysis of patient subclones indicates that those with Amp1q have increased expression of *PHF19*

**Fig. 6:**
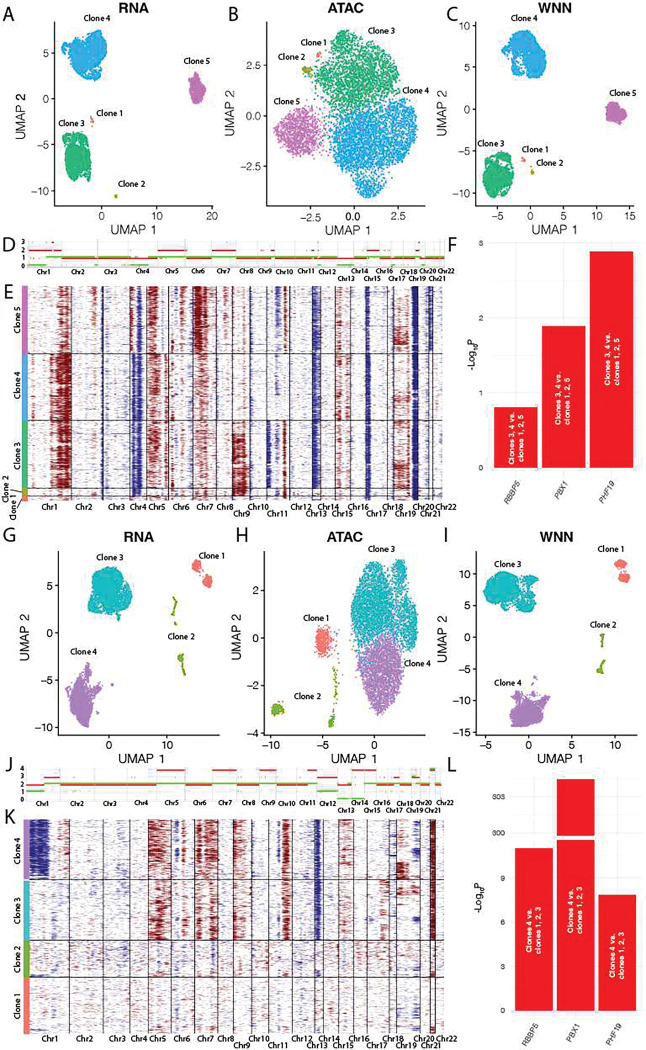
Subclonal differences in Gain/Amp1q. **A-C)** Patient sample 0661–533 clustering based on RNA **(A)**, ATAC **(B)**, WNN integrated RNA and ATAC **(C)**. **D)** WGS based CNV profile. **E)** CNVs inferred from scRNA-seq data. **F)** Increased expression of *PBX1*, *RBBP5*, and *PHF19* in Amp1q clones. **G-I)** Patient sample 0661–1043 clustering based on RNA **(G)**, ATAC **(H)**, WNN integrated RNA and ATAC **(I)**. **J)** WGS based CNV profile. **K)** CNVs inferred from scRNA-seq data. **L)** Increased expression of *PBX1*, *RBBP5*, and *PHF19* in Amp1q clones.

To determine if we could identify the same co-expression in patient samples, we identified patients with subclones of Amp1q to determine if that subclone also had increased expression of *PHF19*. In the first patient (Fig. 6A–F), five clusters were identified from RNA (Fig. 6A), ATAC (Fig. 6B), and WNN integration of RNA and ATAC (Fig. 6C). Based on WGS, this patient had Amp1q and hyperdiploidy (Fig. 6D), and major subclones could be distinguished based on inferred copy number of the scRNA-seq data (Fig. 6E) with 3/5 subclones representing the majority of the cells. Clones 3 and 4 both had Amp1q in contrast to clones 1, 2, and 5 (Fig. 6E). *RBBP5*, *PBX1*, and *PHF19* were all significantly increased in expression in the clones with Gain/Amp1q (Fig. 6F) indicating that subclonal differences of Amp1q could affect *PHF19* expression through increased expression of the key TFs.

A second patient also contained subclonal heterogeneity represented by four clones that could be distinguished by clustering of RNA (Fig. 6G), ATAC (Fig. 6H), and WNN integration of RNA and ATAC (Fig. 6I). Based on WGS, this patient had Amp1q, and was hyperdiploid (Fig. 6J). Clone 4 had Amp1q (Fig. 6K) and also has significantly increased expression of *RBBP5*, *PBX1*, and *PHF19* compared to clones 1, 2, and 3 (Fig. 6L). These examples demonstrate that even at the subclonal level there is likely regulation of *PHF19* by TFs on 1q, leading to high-risk disease.

## DISCUSSION

We report on a large single-cell multiomic study from patients across different stages of disease progression. Based on these data we have identified multiple proliferative clusters that are associated with specific genomic events and stages of myeloma progression. Notably, four such clusters denoted (RRPC) significantly increase in proportion from SMM to RRMM and a subset of three (RRPC_11,20,22_) are associated with amp1q and express higher levels of *PHF19*. The largest subset of RRPCs, RRPC_11_, are enriched predominantly in RRMM patients. RRPC_11_ have increased expression of *PHF19*, a prognostic marker for myeloma, resulting in reduce chromatin accessibility and gene expression of *CDKN1C*. Not surprisingly these same cells exhibit a proliferation signature suggesting a regulatory role of *PHF19* in RRMM patients that results in a more proliferative state. Besides the clear association of RRPCs with myeloma progression, a synergistic association with *TP53* and 1q CNVs was identified in these RRPCs. The TFs *PBX1*, *RFX5*, and *RBBP5* were identified on 1q and may regulate PHF19 based on KD experiments. RRPCs have consistent upregulation of these TFs and PHF19 can also be identified in subclones containing 1q alterations. Taking all of this into consideration this data may indicate an association between *PHF19* and 1q via TF regulation creating unique molecular subtypes of myeloma cells.

Molecular subtypes of myeloma have been identified using analysis of gene expression data including a proliferation (PR) group with worse overall survival and progression free survival than other groups.^2^ At the single cell level, proliferative subpopulations of abnormal plasma cells have been identified in myeloma patients showing that the proliferation signature is not uniformly distributed across plasma cells from a myeloma patient^33^ and that they are more prevalent in relapsed patients.^34^ Furthermore, proliferation was found to be the central prognostic factor for myeloma patients and Gain/Amp1q is highly correlated with higher proliferation index.^11^

Given the high correlation between Gain/Amp1q and the proliferation signature, it is not surprising that Gain/Amp1q is a high-risk cytogenetic event in myeloma. Gain/Amp1q is the most important single event that confers higher risk of relapse.^35^ Our results again highlight that the highest risk subclones with a proliferation signature were highly enriched for Gain/Amp1q. Further study on 1q is needed due to 1q alterations being clearly associated with worse progression, having an incompletely understood mechanism, and the risk of relapse greatly increases in patients with both a 1q alternation and TP53 mutations.^36^ It is of the utmost importance to study mechanistically how 1q is conferring risk and also why there is synergy between 1q alterations, TP53 mutations, and proliferative cellular phenotypes. One such mechanism that has been proposed is the upregulation of the TF PBX1 on 1q23 which causes upregulation of FOXM1.^11^ Interestingly, we find there is also evidence that PHF19 is simultaneously targeted by PBX1 in Gain/Amp1q patients. Considering recent studies have also determined that PHF19 expression is one of the most important single prognostic factors in myeloma,^13^ regulation of PHF19 by 1q alterations would add some context to Gain/Amp1q mechanistically.

Further complicating the relationships between high-risk cytogentic events and progression, is clonal heterogeneity. Based on combined B-cell receptor V(D)J and RNA sequencing, multiple subclones were been identified form NDMM and RRMM patient samples with some convergent proliferative phenotypes associated with progression.^37^ Frequently subclones with Gain/Amp1q tended to have a survival advantage during treatment and leading to an expansion of Gain/Amp1q subclones.^38^ These survival advantages of specific subclones during treatment and relapse, also have an epigenetic regulatory component conferring resistance to therapy.^39^ These recent results further support our findings linking high-risk copy number changes to epigenetic regulation of proliferation pathways.

In summary we have generated one of the largest single cell multiomic datasets for myeloma. From these data we have identified distinct proliferation states that are associated with high-risk cytogenetic events, identified three new TFs to study as they relate to 1q regulation of cell cycle, and discovered a strong link between Amp1q, *PHF19* expression, and proliferation. Specifically, *PHF19* likely epigenetically regulates cell cycle in a subset of cells found primarily in RRMM patients. Gain and especially amplification of PBX1 on 1q may contribute to this high *PHF19* expression phenotype found in these cells.

## Figures and Tables

**Table 1:** Patient Demographics. The following abbreviations were used in the table: smoldering multiple myeloma (SMM), newly diagnosed multiple myeloma (NDMM), relapsed or refractory multiple myeloma (RRMM), copy number variation (CNV), loss of heterozygosity (LOH), mutation (Mut), and hyperdiploidy (HRD).

Diagnosis	SMM	NDMM	RRMM	All
**N**=	10	22	17	49
**Age >65 (%)**	5 (50%)	14 (64%)	11 (65%)	30 (61%)
**Sex (% male)**	4 (40%)	11 (50%)	13 (76%)	21 (43%)
**Translocations**				
**t(4;14) (n/%)**	2 (20%)	1 (5%)	2 (12%)	5 (10%)
**t(6;14)**	0 (0%)	1 (5%)	0 (0%)	1 (2%)
**t(11;14)**	1 (10%)	5 (23%)	4 (24%)	10 (20%)
**t(14;16)**	0 (0%)	0 (0%)	1 (6%)	1 (2%)
**t(14;20)**	0 (0%)	1 (5%)	1 (6%)	2 (4%)
**Hyperdiploidy**	7 (70%)	15 (68%)	8 (47%)	30 (61%)
**CNV**				
**del(CDKN2C)/1p**	0 (0%)	6 (27%)	3 (18%)	9 (18%)
**gain(CKS1B)/1q**	6 (60%)	8 (36%)	6 (35%)	29 (59%)
**amp(CKS1B)/1q**	0 (0%)	4 (18%)	5 (29%)	9 (18%)
**del(RB1)/13q**	6 (60%)	10 (45%)	11 (65%)	27 (55%)
**del(TP53)/17p**	0 (0%)	2 (9%)	5 (29%)	7 (14%)
**Mutations** ^†^				
***TENT5C***	0 (0%)	1 (5%)	1 (7%)	2 (5%)
***TP53***	0 (0%)	2 (10%)	6 (40%)	8 (18%)
***BRAF***	0 (0%)	1 (5%)	2 (13%)	3 (7%)
***NRAS***	1 (11%)	4 (20%)	3 (20%)	8 (18%)
***KRAS***	2 (22%)	5 (25%)	2 (13%)	9 (20%)

† Note that undetermined samples were excluded from the percentage calculation resulting in SMM (N=9), NDMM (N=20), and RRMM (N=15)

**Table 2:** Simplified table categorizing plasma cell clusters.

Category	Cluster
**Normal Plasma Cells (NPCs)**	18
**Hyperdiploid Plasma Cells (HDPCs)**	13
**Malignant Plasma Cells (MPCs)**	1,2,3,4,5,6,7,8,9,10,12,14,15,16,17,19,21,23,24,25
**Relapse/Refractory Plasma Cells (RRPCs)**	11,20, 22, 23

**Table 3:** Table with defining characteristics of plasma cell clusters

PMPC Cluster	
**Cluster 11 (RRPC_11_)**	Gain/Amp1q(*CKS1B*), Amp1q(*CSK1B*), Del12p(*CDKN1B*), Mut(*NRAS*), Mut(*TP53*), Mut(*HUWE1*)
**Cluster 13 (HDPCs)**	HRD, Gain/Amp(*RAPGEF5*), Gain/Amp(*KLF14*), Gain/Amp(*ATM*) Gain/Amp(*WDR72*), Gain/Amp(*BLM*) Gain/Amp(*ZNF426*)
**Cluster 18 (NPCs)**	None
**Cluster 20 (RRPC_20_)**	Gain/Amp1q(*CKS1B*), Amp1q(*CSK1B*), Del12p(*CDKN1B*), Mut(*TP53*), Mut(*USP7*), Mut(*HUWE1*)
**Cluster 22 (RRPC_22_)**	Gain/Amp1q(*CKS1B*), Amp1q(*CSK1B*), Gain/Amp(*RAPGEF5*), Gain/Amp(*KLF14*), Gain/Amp(CCND1), Gain/Amp(ATM)
**Cluster 23 (RRPC_23_)**	Gain/Amp1q(*CKS1B*)

## Data Availability

Data are available through dbGAP at accession number phs003220.
